# 在线固相萃取-超高效液相色谱-三重四极杆质谱法测定水源水和饮用水中107种典型农药及代谢产物

**DOI:** 10.3724/SP.J.1123.2022.07011

**Published:** 2022-12-08

**Authors:** Yongyan CHEN, Jia LÜ, Lan ZHANG, Bixiong YE, Ning JIN

**Affiliations:** 中国疾病预防控制中心环境与人群健康重点实验室, 中国疾病预防控制中心环境与健康相关产品安全所, 北京 100050; China CDC Key Laboratory of Environment and Population Health, National Institute of Environmental Health, Chinese Center for Disease Control and Prevention, Beijing 100050, China

**Keywords:** 超高效液相色谱-三重四极杆质谱, 在线固相萃取, 农药, 水源水, 饮用水, ultra performance liquid chromatography-triple quadrupole mass spectrometry (UPLC-MS/MS), online-solid phase extraction (online-SPE), pesticides, raw water, drinking water

## Abstract

为进行我国水体中农药风险监测,针对水体中农药种类多、浓度低的特点,建立了在线固相萃取-超高效液相色谱-串联质谱法快速筛查和检测水源水及饮用水中107种典型农药及代谢产物(有机磷类、有机氮类、有机杂环类、氨基甲酸酯类、酰胺类、苯甲酰脲类、新烟碱类等)的方法。样品经0.22 μm孔径亲水性聚四氟乙烯滤膜过滤后,通过自动进样器取5 mL样品注入在线固相萃取系统,经X Bridge C_18_在线固相萃取柱吸附后用纯水淋洗,以乙腈和0.1%甲酸水溶液为流动相对在线固相萃取柱梯度洗脱后再经ACQUITY HSS T_3_色谱柱分离,采用电喷雾离子源正离子及负离子模式分析检测,外标法定量。以水源水及饮用水作为基质,对其准确度和精密度进行方法学验证,结果表明:107种农药及代谢产物在不同范围内线性关系良好(*r*^2^>0.995),方法检出限(LOD, *S/N*=3)为0.03~1.5 ng/L,定量限(LOQ, *S/N*=10)为0.1~5.0 ng/L。将目标分析物在1、20、50 ng/L水平下加标,水源水和饮用水中的加标回收率分别为60.6%~119.8%和61.2%~119.0%,相对标准偏差(RSD, *n*=6)分别为0.3%~18.6%和0.4%~17.1%。用该方法测定水源水和饮用水中的农药残留,结果显示,酰胺类、三嗪类除草剂、三唑类杀菌剂与烟碱类、氨基甲酸酯类杀虫剂有较高的检出率,其中水源水中检出含量为0.1~97.1 ng/L,饮用水中检出含量为0.1~93.6 ng/L。该方法适用于水源水和饮用水中107种典型农药及代谢产物的痕量分析测定,有效提高了水体中农药类物质的检测效率,实际应用价值较高。

## Abstract (en)

In order to monitor the risk of pesticide pollutants in drinking water, an analytical method based on online-solid phase extraction coupled with ultra performance liquid chromatography-triple quadrupole mass spectrometry (online-SPE-UPLC-MS/MS) was established for the simultaneous rapid screening and determination of 107 pesticides and metabolites (organophosphorus, organic nitrogen, organic heterocycle, carbamate, amide, benzoyl urea, neonicotinoid, etc.) in raw water and drinking water. Different injection volumes (5, 10, and 15 mL) were compared. The detection response increased with an increase in the injection volume, but the matrix effect also became more pronounced. Under the premise of ensuring the sensitivity of the method and meeting the detection requirements, the injection volume was selected as 5 mL. Accordingly, the samples were filtered through a 0.22-μm hydrophilic polytetrafluoroethylene filter, and then, 5 mL samples were injected into the online-SPE system by the automatic sampler. After adsorption on an X Bridge C_18_ online-SPE column, the samples were washed with pure water and eluted by gradient elution using acetonitrile and 0.1% formic acid aqueous solution as the mobile phases, with separation on an ACQUITY HSS T_3_ column. The samples were detected by multiple reaction monitoring with electrospray ionization in positive and negative ion modes, and quantified by an external standard method. Using raw water and drinking water as the sample matrices, the accuracy and precision of the method were verified. The 107 pesticides and metabolites showed good linear relationships in different ranges with correlation coefficients (*r*^2^)>0.995. The limits of detection (LODs, *S/N*=3) of the method were 0.03-1.5 ng/L, and the limits of quantification (LOQs, *S/N*=10) were 0.1-5.0 ng/L. The target pesticides were spiked at concentration levels of 1, 20, and 50 ng/L. The spiked recoveries of the 107 targets in raw water and drinking water samples were 60.6%-119.8% and 61.2%-119.0%, respectively. The corresponding relative standard deviations (RSDs, *n*=6) were 0.3%-18.6% and 0.4%-17.1%. The pesticide residues in raw water and drinking water were determined by this method. Amide herbicides, triazine herbicides, triazole insecticides, carbamate insecticides, and neonicotinoid insecticides had high detection rates. The detected concentrations ranged from 0.1 to 97.1 ng/L in raw water and from 0.1 to 93.6 ng/L in drinking water. The sample consumption of online-SPE method was lower than that in the traditional off-line SPE methods, which greatly improved the convenience of sample collection, storage, and transportation. The samples only need to be filtered before injection and analysis. The method is simple to operate and shows good reproducibility. With this online-SPE method, only 23 min were required from online enrichment to detection completion. The developed method has the advantages of high analytical speed and high sensitivity. The method is suitable for the trace analysis and determination of 107 typical pesticides in raw water and drinking water, which effectively improves the detection efficiency of pesticides in water and has high potential for practical application. It can extend technical support for the pollution-level analysis of typical pesticides and metabolites in drinking water and provide an objective basis for human health risk assessment.

我国是农药生产和使用大国。2020年我国化学农药原药总产量214.80万吨^[1]^, 2020年全国农药品种数量714个,比2010年增加97个。农药登记产品总数41885个,比2010年增加12688个^[2]^。在农药生产及使用过程中,农药通过地表径流、扩散等途径进入环境水体中,并在迁移过程中降解转化为新的代谢产物,势必在水源水及饮用水中存在农药及其代谢产物的暴露风险^[3⇓-5]^。水体中农药复合污染情况较为普遍,同时具有种类多、浓度低的特点^[6⇓⇓⇓-10]^。针对农药类污染物进行风险监测,开发高灵敏度、多指标同时测定的方法很有必要。本研究结合国内外饮用水标准中相关农药指标及我国用量较大的农药类化合物进行遴选^[11]^,建立了在线固相萃取-超高效液相色谱-串联质谱法(online-SPE-UPLC-MS/MS)快速筛查和检测水源水及饮用水中107种典型农药及代谢产物的方法,可为开展水体中典型农药及代谢产物的污染水平分析提供技术支持,为人体健康风险评估提供客观依据。

目前国内外水体中农药的相关检测方法主要基于色谱或色谱与质谱联用技术^[12⇓-14]^。虽然液相色谱仪及气相色谱仪普及率较高,但其检测方法灵敏度相对较低,定性能力较差。气相色谱-质谱法对于水样的前处理要求较液相色谱-质谱法(LC-MS)苛刻,LC-MS具有高灵敏度、高通量以及假阳性率较低等优点,是农药检测的重要手段。水体中农药类污染物赋存水平低,直接进样法虽然便捷快速,但难以满足部分农药类物质的灵敏度需求,需富集后测定。常用的提取、富集技术有液液萃取、固相萃取、固相微萃取等,但前处理耗时往往长于仪器分析检测时间,制约检测效率。在线SPE技术与离线SPE相比,上样时间由1~2 h缩短到几分钟,水样体积由几百毫升降低至几毫升,样品采集、运输及保存的便捷性大大提升。同时由于自动化程度高,人员操作步骤少,实验操作误差小。本研究基于该前处理技术建立了适用于水源水及饮用水中107种典型农药及其代谢产物的检测方法,从样品富集至检测完成仅耗时20余分钟,检测用时短、灵敏度高、重复性好,可用于水源水及饮用水中典型农药类污染物检测及风险监测。

## 1 实验部分

### 1.1 仪器、试剂与材料

ACQUITY UPLC-XEVO Micro TQS超高效液相色谱-串联质谱和OA system全自动在线前处理系统购自美国Waters公司;乙腈、甲醇(德国Merck公司);甲酸(HPLC-MS级,美国Fisher公司);抗坏血酸(分析纯,阿拉丁试剂有限公司); 0.22 μm亲水性聚四氟乙烯滤膜(美国Pall公司); 107种农药及代谢物混合标准溶液(100 μg/mL,溶剂为乙腈,天津阿尔塔科技有限公司)。

### 1.2 标准溶液的配制

100 μg/L混合标准储备液:准确移取100 μL 100 μg/mL的107种混合标准溶液至100 mL容量瓶中,用乙腈定容后混匀,-18 ℃避光保存备用。

0.1 μg/L混合标准溶液:准确移取100 μL 100 μg/L的混合农药标准溶液至100 mL容量瓶中,用纯水定容后混匀,现用现配。

标准溶液系列:准确移取一定量的0.1 μg/L混合标准溶液,以纯水为溶剂,分别配制质量浓度为0.1、0.2、0.5、1.0、2.0、5.0、20、50、100 ng/L的混合标准溶液,现用现配。

### 1.3 样品采集

使用棕色螺口玻璃瓶进行样品采集,瓶盖含有聚四氟乙烯内衬垫片,满瓶采样。采样时每升样品中添加50 mg抗坏血酸,4 ℃冷藏避光保存至进样分析。

### 1.4 样品在线净化富集

在线SPE系统为两根SPE柱平行设计,如图1所示,本研究使用两根X Bridge C_18_ SPE柱,分别与二元泵(pump 1)和四元泵(pump 2)连接。一根SPE柱在四元泵系统进样富集后通过阀切换至二元泵系统,通过流动相将目标分析物洗脱入分析柱分离后进行质谱检测,同时另一根SPE柱同步进行阀切换,由二元泵系统通过切换至四元泵系统进行再生,以备下一样品进样、富集使用。由此往复,实现SPE柱交替使用,节约了再生、平衡时间。

**图1:**
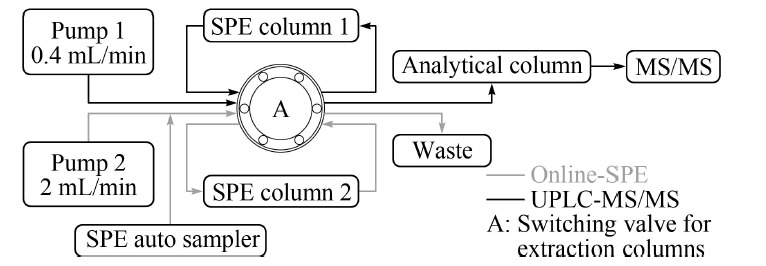
在线固相萃取系统示意图

### 1.5 仪器条件

在线SPE:流动相A为纯水;流动相B为乙腈;在线SPE柱为X Bridge C_18_(30 mm×2.1 mm, 10 μm,美国Waters公司);进样量:5 mL。梯度洗脱程序见表1,其中3.5~4.5 min为阀切换时间。

**表1:** 在线固相萃取梯度洗脱条件

Time/ min	Flow rate/ (mL/min)	φ(Water)/ %	φ(Acetonitrile)/ %	Curve parameter
0	2.00	100	0	11^*^
0.5	1.00	100	0	11
3.5	0.01	100	0	11
4.5	2.00	0	100	11
7.5	2.00	100	0	11
13.0	2.00	100	0	11
23.0	2.00	100	0	11

11^*^: keep the starting proportion and flow rate unchanged before the next step.

液相色谱分离:流动相A为0.1%甲酸水溶液;流动相B为乙腈;分析柱为ACQUITY UPLC HSS T_3_(100 mm×2.1 mm, 1.8 μm,美国Waters公司);柱温40 ℃。梯度洗脱程序见表2,其中3.5~4.5 min为阀切换时间。

**表2:** 液相色谱梯度洗脱条件

Time/ min	Flow rate/ (mL/min)	φ(0.1%FA)/ %	φ(Acetonitrile)/ %	Curve parameter
0	0.40	98	2	11^*^
3.5	0.01	98	2	11
4.5	0.40	98	2	11
5.0	0.40	98	2	6^*^
17.0	0.40	5	95	6
20.0	0.40	5	95	6
20.2	0.40	98	2	6
23.0	0.40	98	2	6

FA: formic acid aqueous solution. 6^*^: linear; 11^*^: keep the starting proportion and flow rate unchanged before the next step.

### 1.6 质谱条件

电喷雾离子源,正离子模式及负离子模式扫描,数据采集方式为多反应监测(MRM);去溶剂气:高纯氮气,去溶剂温度500 ℃,流量1000 L/h。锥孔气:高纯氮气,流量50 L/h。离子源温度150 ℃,碰撞气为氩气,毛细管电压1.5 kV。107种农药及代谢产物的离子对及质谱采集参数见表3。

**表3:** 目标分析物的保留时间与质谱参数

No.	Pesticide	ESI	Retention time/min	Ion pairs (m/z)	CV/V	CEs/eV
1	abamectin (阿维菌素)	+	16.9	890.5>305.2, 890.5>567.3	82	26, 15
2	acetamiprid (啶虫脒)	+	9.6	223.0>125.8, 223.0>98.9	24	20, 38
3	acetochlor (乙草胺)	+	13.8	270.1>224.0, 270.1>148.1	32	6, 16
4	acifluorfen (三氟羧草醚)	-	13.8	360.0>316.0, 360.0>195.0	15	10, 24
5	alachlor (甲草胺)	+	13.8	270.1>238.0, 270.1>162.2	30	10, 20
6	aldicarb (涕灭威)	+	10.3	213.1>89.0, 213.1>115.9	30	18, 12
7	aldicarb sulfone (涕灭威砜)	+	8.1	223.0>86.0, 223.0>147.9	42	12, 8
8	anilofos (莎稗磷)	+	14.4	368.1>198.9, 368.1>124.9	28	12, 32
9	atrazine (莠去津)	+	11.6	216.1>174.0, 216.1>103.9	36	15, 28
10	atrazine-2-hydroxy (2-羟基莠去津)	+	8.1	198.1>85.9, 198.1>113.9	34	24, 22
11	atrazine-desethyl (脱乙基莠去津)	+	9.4	188.0>145.9, 188.0>103.9	36	18, 24
12	atrazine-desisopropyl (脱异丙基莠去津)	+	8.5	174.0>95.9, 174.0>103.9	76	16, 20
13	beauvericin (白僵菌素)	+	17.1	784.4>134.0, 784.4>244.1	50	62, 28
14	benfuracarb (丙硫克百威)	+	15.7	411.2>195.0, 411.2>252.0	28	24, 14
15	benzovindiflupyr (苯并烯氟菌唑)	+	14.3	398.1>342.1, 398.1>378.1	32	16, 12
16	buprofezin (噻嗪酮)	+	14.6	306.1>201.1, 306.1>115.9	24	10, 14
17	butachlor (丁草胺)	+	15.8	312.2>238.0, 312.2>162.1	36	12, 21
18	cadusafos (硫线磷)	+	14.6	271.0>158.9, 271.0>130.8	32	14, 24
19	carbaryl (甲萘威)	+	11.5	202.1>145.0, 202.1>126.9	24	6, 24
20	carbendazim (多菌灵)	+	7.6	192.0>160.0, 192.0>132.0	48	16, 26
21	carbofuran (克百威)	+	11.2	222.1>122.9, 222.1>165.0	32	18, 12
22	chlorobenzuron (灭幼脲)	+	13.8	309.0>156.0, 309.0>138.9	38	14, 29
23	chlorotoluron (绿麦隆)	+	11.4	213.0>71.9, 213.0>45.9	46	18, 14
24	chlorpyrifos (毒死蜱)	+	15.9	350.0>197.9, 350.0>96.9	20	16, 28
25	chlorpyrifos-methyl (甲基毒死蜱)	+	14.9	321.9>124.8, 321.9>289.8	32	20, 10
26	chlorsulfuron (氯磺隆)	+	11.4	358.0>141.0, 358.0>167.0	30	16, 14
27	clothianidin (噻虫胺)	+	9.1	250.0>168.9, 250.0>132.0	22	10, 14
28	coumaphos (蝇毒磷)	+	14.7	363.0>226.9, 363.0>307.0	40	24, 14
29	cyanazine (氰草津)	+	10.8	241.1>214.0, 241.1>103.9	40	16, 28
30	demeton O & S (内吸磷)	+	12.2	259.0>88.9, 259.0>60.9	10	10, 35
31	demeton-S-methyl sulfone (内吸磷-S-甲基-砜)	+	8.6	263.0>168.9, 263.0>124.9	40	14, 23
32	demeton-S-sulfoxide (内吸磷-S-亚砜)	+	9.1	275.0>197.0, 275.0>140.9	30	12, 22
33	dichlorvos (敌敌畏)	+	10.8	220.9>108.9, 220.9>144.9	42	14, 10
34	diflubenzuron (除虫脲)	+	13.5	311.0>157.9, 311.0>140.9	25	12, 32
35	dimethoate (乐果)	+	9.6	230.0>198.9, 230.0>124.8	28	8, 20
36	dinocap (敌螨普)	-	16.4	295.0>209.0, 295.0>134.0	30	28, 55
37	dinoseb (地乐酚)	-	14.3	239.0>194.1, 239.0>134.0	54	20, 46
38	disulfoton-sulfone (乙拌磷砜)	+	12.5	307.0>124.8, 307.0>152.9	30	16, 10
39	disulfoton-sulfoxide (乙拌磷亚砜)	+	11.5	291.0>184.9, 291.0>212.9	32	12, 8
40	diuron (敌草隆)	+	11.8	233.0>71.9, 233.0>45.9	32	18, 14
41	EPN (苯硫磷)	+	15.1	324.0>296.0, 324.0>156.9	14	12, 22
42	fenamiphos (苯线磷)	+	13.1	304.0>216.9, 304.0>201.9	50	22, 32
43	fenamiphos sulfone (苯线磷砜)	+	11.1	336.1>266.0, 336.1>308.0	45	18, 18
44	fenamiphos sulfoxide (苯线磷亚砜)	+	10.3	320.1>107.9, 320.1>171.0	42	40, 25
45	fenobucarb (仲丁威)	+	12.7	208.1>94.9, 208.1>151.8	22	12, 8
46	fenthion (倍硫磷)	+	14.4	279.0>168.9, 279.0>246.9	40	16, 10
47	fenthion sulfoxide (倍硫磷亚砜)	+	11.2	295.0>108.9, 295.0>279.9	48	30, 16
48	fenthion-sulfone (倍硫磷砜)	+	12.2	311.0>124.8, 311.0>109.0	44	22, 28
49	fipronil (氟虫腈)	-	14.2	435.0>329.9, 435.0>249.9	30	14, 26
50	fipronil desulfinyl (氟甲腈)	-	14.5	386.9>350.9, 386.9>281.9	32	10, 30
51	fipronil sulfide (氟虫腈硫化物)	-	14.8	418.9>382.9, 418.9>261.9	30	10, 28
52	fipronil-sulfone (氟虫腈砜)	-	14.7	450.9>414.9, 450.9>282.0	54	16, 26
53	fonofos (地虫硫磷)	+	14.7	246.9>108.8, 246.9>136.9	8	16, 10
54	fosthiazate (噻唑磷)	+	11.6	284.0>103.9, 284.0>228.0	20	20, 10
No.	Pesticide	ESI	Retention time/min	Ion pairs (m/z)	CV/V	CEs/eV
55	3-hydroxycarbofuran (3-羟基克百威)	+	9.2	238.1>181.0, 238.1>163.0	38	10, 16
56	imidacloprid (吡虫啉)	+	9.4	256.0>209.1, 256.0>175.0	40	12, 14
57	imidacloprid-urea (吡虫啉尿素)	+	8.8	212.0>127.9, 212.0>98.9	54	16, 18
58	imidaclothiz (氯噻啉)	+	9.6	262.1>181.0, 262.1>122.3	25	14, 28
59	iprobenfos (异稻瘟净)	+	13.6	289.1>90.9, 289.1>205.0	20	18, 8
60	isoprocarb (异丙威)	+	12	194.1>94.9, 194.1>137.0	26	12, 8
61	isoprothiolane (稻瘟灵)	+	13.6	291.0>230.9, 291.0>188.9	30	10, 20
62	isoproturon (异丙隆)	+	11.7	207.1>71.9, 207.1>165.0	42	20, 10
63	malaoxon (马拉氧磷)	+	11.1	315.0>98.9, 315.0>126.9	30	24, 12
64	malathion (马拉硫磷)	+	13.6	331.0>126.9, 331.0>98.9	30	12, 20
65	mefenacet (苯噻酰草胺)	+	13.2	299.1>148.0, 299.1>120.0	42	14, 26
66	mepronil (灭锈胺)	+	13.6	270.1>118.9, 270.1>90.9	46	20, 42
67	metalaxyl (甲霜灵)	+	11.8	280.1>220.0, 280.1>192.1	26	12, 18
68	methidathion (杀扑磷)	+	12.8	302.9>144.9, 302.9>85.0	2	8, 21
69	metolachlor (异丙甲草胺)	+	13.8	284.1>252.1, 284.1>176.1	20	12, 24
70	metsulfuron-methyl (甲磺隆)	+	11.1	382.0>167.0, 382.0>141.0	26	14, 12
71	molinate (禾草敌)	+	13.1	188.0>126.0, 188.0>54.9	32	12, 22
72	monocrotophos (久效磷)	+	8.2	224.0>126.9, 224.0>192.9	32	14, 6
73	nitenpyram (烯啶虫胺)	+	8.2	271.2>225.1, 271.2>99.0	32	10, 16
74	oxamyl (杀线威)	+	8.2	237.0>71.9, 237.0>89.9	10	12, 6
75	parathion (对硫磷)	+	14.4	291.9>235.9, 291.9>93.9	32	14, 34
76	parathion-methyl (甲基对硫磷)	+	13.2	264.0>124.8, 264.0>108.8	42	16, 24
77	pencycuron (戊菌隆)	+	14.8	329.1>124.9, 329.1>218.0	20	22, 16
78	pendimethalin (二甲戊灵)	+	15.9	282.0>212.0, 282.0>194.1	28	10, 18
79	phenthoate (稻丰散)	+	14.5	320.9>163.0, 320.9>135.0	26	10, 22
80	phorate (甲拌磷)	+	14.8	261.0>74.9, 261.0>46.9	6	10, 38
81	phorate sulfone (甲拌磷砜)	+	12.6	293.0>96.9, 293.0>247.0	21	36, 6
82	phorate sulfoxide (甲拌磷亚砜)	+	11.5	277.0>198.9, 277.0>142.9	26	8, 18
83	phosfolan-methyl (甲基硫环磷)	+	8.6	228.0>167.9, 228.0>108.8	22	12, 24
84	phosphamidon (磷胺)	+	10.4	300.0>174.0, 300.0>126.9	46	12, 20
85	phoxim (辛硫磷)	+	14.8	299.0>128.9, 299.0>152.9	8	12, 6
86	pretilachlor (丙草胺)	+	15.2	312.1>252.0, 312.1>176.1	30	16, 28
87	procymidone (腐霉利)	+	13.7	284.0>256.0, 286.0>258.0	50	16, 16
88	pymetrozin (吡蚜酮)	+	5.7	218.0>104.9, 218.0>78.4	32	18, 36
89	pyridaben (哒螨灵)	+	16.7	365.1>147.0, 365.1>309.1	10	24, 12
90	rotenone (鱼藤酮)	+	13.8	395.2>192.1, 395.2>213.1	37	25, 24
91	simazine (西玛津)	+	10.6	202.0>123.9, 202.0>131.9	24	16, 18
92	simetryn (西草净)	+	10.1	214.0>124.0, 214.0>96.1	22	18, 24
93	sulfotep (治螟磷)	+	14.8	323.0>171.0, 323.0>114.8	20	12, 30
94	tebuconazole (戊唑醇)	+	13.4	308.1>70.0, 308.1>124.9	44	20, 38
95	terbufos (特丁硫磷)	+	15.8	288.9>102.9, 288.9>232.9	18	10, 4
96	terbufos sulfone (特丁硫磷砜)	+	13.4	321.0>96.9, 321.0>114.8	46	42, 26
97	terbufos sulfoxide (特丁磷亚砜)	+	12.5	305.0>186.9, 305.0>130.8	16	8, 28
98	terbuthylazine (特丁津)	+	12.7	230.1>174.0, 230.1>104.0	26	14, 32
99	thiabendazole (噻苯哒唑)	+	7.9	202.0>175.0, 202.0>131.0	44	24, 32
100	thiacloprid (噻虫啉)	+	10.2	253.1>125.9, 253.1>185.9	20	18, 12
101	thiamethoxam (噻虫嗪)	+	8.7	292.0>211.0, 292.0>181.1	20	10, 22
102	thiodicarb (硫双威)	+	11.2	355.0>87.9, 355.0>107.8	10	14, 16
103	triadimefon (三唑酮)	+	13.2	294.0>197.0, 294.0>225.0	36	14, 12
104	triazophos (三唑磷)	+	13.7	314.0>162.0, 314.0>119.0	40	18, 34
105	tricyclazole (三环唑)	+	9.7	190.0>136.0, 190.0>163.0	44	26, 22
106	triflumuron (杀铃脲)	+	14.3	359.1>155.9, 359.1>138.9	32	16, 30
107	trifluralin (氟乐灵)	+	11.0	336.1>249.0, 336.1>294.1	30	25, 13

CV: cone voltage; CEs: collision energies. EPN: *O*-ethyl *O*-(4-nitrophenyl) phenylphosphonothioate.

## 2 结果与讨论

### 2.1 色谱条件考察

本研究目标分析物种类多、极性差异大,因此选用通用型反相色谱柱进行色谱分离,以获得较好的色谱保留和色谱峰形。通过对HSS T_3_及BEH C_18_两种色谱柱比较发现,两者均能实现对目标分析物的较好分离,但HSS T_3_色谱柱在反相条件下对于极性较强的化合物保留更强,从而延长了该类物质的保留时间,更适合极性与非极性化合物混合样品的测定,因此选取HSS T_3_色谱柱。本研究中分析物多以ESI^+^模式扫描测定(100种),流动相中加入少量甲酸对化合物电离后加氢具有促进作用,对灵敏度呈正影响,分别对不同体积分数的甲酸水溶液(0.05%、0.1%、0.2%)与乙腈混合的流动相进行考察,同时兼顾ESI^-^模式下分析物(7种)的响应值,最终确定使用0.1%甲酸水-乙腈为流动相。在线SPE系统中流动相先将吸附在SPE柱上的目标物洗脱,后将其带入分析柱色谱分离,本研究对色谱分离程序中不同初始有机相比例对分离效果进行比较,结果表明,初始流动相中乙腈比例为2%效果最佳,在该条件下目标化合物在分析柱上等度洗脱0.5 min后梯度洗脱,乙腈比例梯度上升至95%,可完成目标分析物的完全洗脱和有效分离。

### 2.2 质谱条件优化

根据107种典型农药及代谢产物的化学电离性质,本研究采用正、负电离模式同时采集。以乙腈为溶剂分别配制质量浓度为200 μg/L的标准物质溶液,泵入质谱内进行全扫描分析,分别选取丰度较强的离子作为定性和定量依据。其中甲草胺和乙草胺较为特殊,其为同分异构体,保留时间极为接近,母离子都为*m/z* 270.09,但甲草胺最强特征子离子为*m/z* 238.02和162.08,乙草胺最强特征子离子为*m/z* 244.03和148.06,虽然二者结构类似,但可排除两者碎片离子交叉干扰,见图2。质谱条件优化中,锥孔电压和碰撞能量对灵敏度影响最大,本研究针对目标分析物逐一优化后确定最佳锥孔电压和碰撞能量。毛细管电压对灵敏度也有一定影响,将一定质量浓度的混合标准溶液在毛细管电压0.5、1.5、2.5、3.5 kV的条件下分别进行质谱采集,综合各分析物响应值强度选取1.5 kV为毛细管电压。

**图2:**
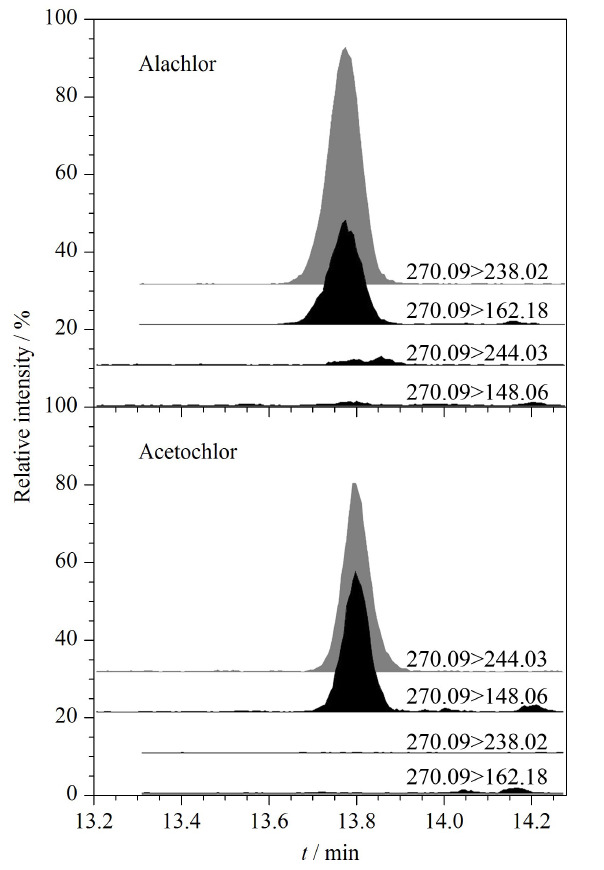
甲草胺和乙草胺的特征离子

### 2.3 在线SPE柱的选择

SPE柱的填料种类决定了其对目标分析物的吸附能力。本研究选取X Bridge C_8_、X Bridge C_18_、Oasis HLB 3种通用型反相吸附SPE柱进行考察。对极性化合物的吸附能力由强到弱分别为Oasis HLB>X Bridge C_18_>X Bridge C_8_,其中X Bridge C_8_的填料与X Bridge C_18_相近,更适用于中等疏水性化合物吸附,因此重点比较了Oasis HLB和X Bridge C_18_的富集、洗脱效果。经流动相梯度洗脱条件优化后,使用两款SPE柱时目标分析物的回收率均可达到60%以上,大部分目标分析物的峰形及响应值能够满足检测需求,少量峰形及响应值差异明显。选取6种差异较大的典型分析物为代表进行比较。对于极性较强的化合物,在Oasis HLB柱上保留更佳,如甲基硫环磷,在Oasis HLB柱上富集后质谱检测响应值是X Bridge C_18_柱的3倍左右,见图3。但对于极性相对较弱的化合物,Oasis HLB柱吸附洗脱后,有色谱峰较宽、分离度差、拖尾等现象,同等条件下X Bridge C_18_柱吸附洗脱后,色谱峰形尖锐、对称,方法精密度优于Oasis HLB柱。综合考虑,本研究选取X Bridge C_18_柱在线富集。

**图3:**
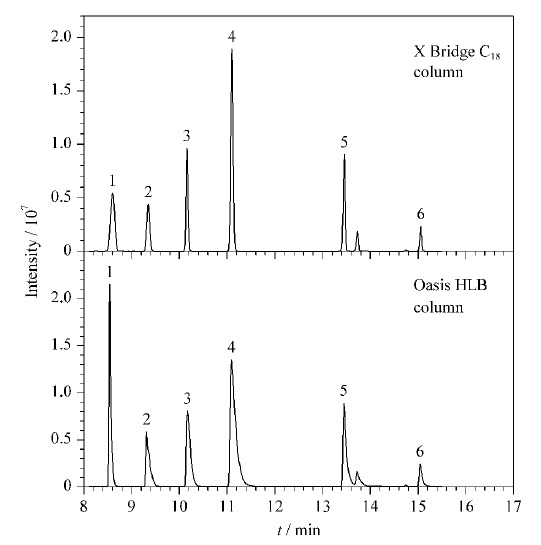
不同在线SPE柱的保留效果

### 2.4 在线固相萃取条件的优化

在线SPE过程与传统的离线SPE过程类似,不同上样、淋洗、洗脱条件对目标分析物的峰形、方法灵敏度都有关键影响。综合各分析物峰形、灵敏度及回收率,实验发现在中性条件下以纯水将样品带入SPE柱,并使用纯水为淋洗液效果最佳。一方面实验中发现部分物质如甲胺磷、辛硫磷等在碱性条件中不稳定,丙硫克百威等酸性条件下易降解,中性条件下相对稳定。另一方面使用酸性或碱性水溶液淋洗易把SPE柱上保留较弱的目标分析物解吸,因此本研究选取中性条件下上样、淋洗。

实验对不同的进样体积(5、10和15 mL)进行比较,结果表明进样体积增大,检测响应值随之提升,但基质效应也会更为明显。本研究以回收率衡量基质效应产生的影响,除了马拉氧磷、腐霉利、白僵菌素大体积进样后产生基质增强效应外,其他分析物均受到不同程度的基质抑制,见图4。因此在确保方法灵敏度满足检测要求的情况下,本研究选取进样体积为5 mL。

**图4:**
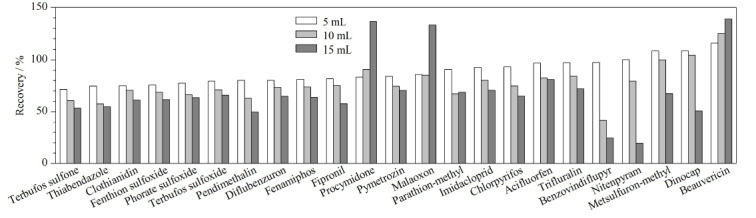
进样体积对回收率的影响

### 2.5 样品采集及预处理条件优化

#### 2.5.1 样品中余氯对农药测定的影响

消毒是生活饮用水处理工艺中的一个重要步骤,《生活饮用水卫生标准》(GB 5749-2022)对总氯、游离氯均有限值要求,例如要求游离氯余量出厂水≥0.3 mg/L,末梢水≥0.05 mg/L^[15]^。为了准确测定饮用水中农药类物质残留,本研究进行了余氯对农药类分析物测定的影响实验。结果表明,107种目标分析物指标中约50%的目标分析物与余氯发生不同程度的氧化还原反应。在未加入抗坏血酸进行脱氯的样品中,氟虫腈、苯线磷、杀铃脲、除虫脲、地虫硫磷、甲拌磷、苯硫磷、倍硫磷、特丁硫磷、烯啶虫胺、莎稗磷、多菌灵、西草净、涕灭威、乐果、治螟磷、甲拌磷亚砜、稻丰散、杀扑磷、乙拌磷砜、蝇毒磷、特丁磷亚砜、丙硫克百威、灭幼脲、内吸磷、乙拌磷亚砜回收率均低于10%,受余氯影响最为突出。其他农药物质受余氯影响发生不同程度的降解或转化,如马拉氧磷为马拉硫磷的代谢产物,苯线磷亚砜为苯线磷的代谢产物,余氯的存在促使了农药与其代谢产物的转化。样品中添加抗坏血酸去除余氯后,样品回收率及稳定性明显提高,见图5。本研究同时对样品中抗环血酸的添加量进行实验,分别按照每升样品中添加20、50、100 mg进行对比,实验结果表明,以上3个抗坏血酸添加量对回收率没有显著性影响。因此,为确保充分消除余氯干扰,本研究在样品采集时,每升样品中添加50 mg抗坏血酸。

**图5:**
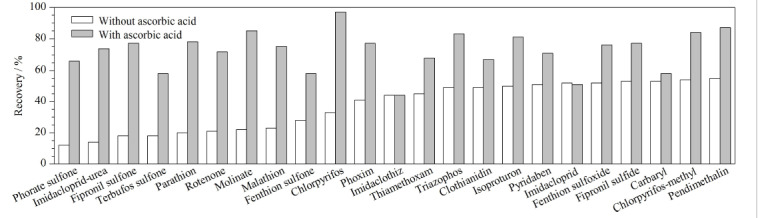
抗坏血酸消除余氯对回收率的影响

#### 2.5.2 滤膜吸附对农药测定的影响

本研究选用针头式微滤膜过滤器对样品进行过滤处理,通过对亲水性聚四氟乙烯滤膜、玻璃纤维素滤膜、尼龙滤膜、聚醚砜滤膜进行比较发现,不同材质的滤膜对农药类分析物的吸附效应不同,亲水性聚四氟乙烯滤膜对农药类分析物的吸附效应最弱,适合用于样品进样分析前过滤处理。针对亲水性聚四氟乙烯滤膜进一步考察了过滤体积对农药类分析物吸附效应的影响。以10 mL为间隔,分段收集A (0~10 mL)、B (10~20 mL)、C (20~30 mL)、D (30~40 mL) 4组过滤液进行试验对比发现,在过滤初始阶段吸附效应最为明显,A组滤液中107种农药类分析物的平均吸附率为17.1%,其中26种农药类分析物吸附率大于20%;随着过滤体积的增加,B组,C组、D组滤液的吸附率显著低于A组,平均吸附率分别为5.5%、5.6%、4.9%,组间无显著性差异,且滤液中99种农药类分析物吸附率小于10%。以上结果表明,增加过滤体积可以减少吸附效应,因此本研究选取舍弃初滤液10 mL,以降低吸附效应、提高实验准确性。

### 2.6 方法学考察

#### 2.6.1 线性关系和检出限

按照1.2节描述配制不同浓度的混合标准溶液,标准溶液经在线SPE处理后测定,以各分析物的质量浓度为横坐标,定量离子的响应值为纵坐标绘制标准曲线。实验结果表明,107种农药及其代谢产物在各自的浓度范围内线性关系良好(相关系数(*r*^2^)>0.995)。以各目标分析物3倍和10倍信噪比(*S/N*)对应的质量浓度确定本方法的检出限(LOD, *S/N*=3)和定量限(LOQ, *S/N*=10),见表4,方法定量限均低于《生活饮用水卫生标准》(GB 5749-2022)、WHO《饮用水水质准则》等国内外饮用水标准中农药类指标相关的限值。

**表4:** 107种目标分析物的线性范围、相关系数、检出限、定量限、加标回收率及RSD(*n*=6)

No.	Pesticide	Linear range/ (ng/L)		LOD/ (ng/L)	LOQ/ (ng/L)	r^2^	Raw water											
							Content/ (ng/L)	Spiked at 1 ng/L			Spiked at 20 ng/L			Spiked at 50 ng/L				
								Recovery/ %	RSD/ %		Recovery/ %	RSD/ %		Recovery/ %	RSD/ %			
1	abamectin	5	-100	1.5	5.0	0.9953	ND	64.6^*^	6.5		61.1	9.1		61.7	9.6
2	acetamiprid	0.2	-100	0.06	0.2	0.9999	0.2	86.3	1.7		92.6	1.4		90.5	1.0
3	acetochlor	1	-100	0.3	1.0	0.9995	1.6	95.1	7.0		96.7	2.1		92.1	1.8
4	acifluorfen	1	-100	0.3	1.0	0.9993	ND	93.4	14.6		94.4	3.1		90.6	1.0
5	alachlor	0.1	-100	0.03	0.1	0.9999	ND	88.7	3.6		98.4	2.1		94.8	1.9
6	aldicarb	5	-100	1.5	5.0	0.9976	ND	61.5^*^	7.0		95.7	9.0		82.3	8.7
7	aldicarb sulfone	5	-100	1.5	5.0	0.9959	ND	88.0^*^	8.6		72.5	10.6		72.6	10.2
8	anilofos	0.1	-100	0.03	0.1	0.9998	ND	90.8	2.6		105.1	4.2		99.4	0.5
9	atrazine	0.1	-100	0.03	0.1	0.9999	8.3	67.1	6.4		103.5	1.1		101.8	0.6
10	atrazine-2-hydroxy	0.1	-100	0.03	0.1	0.9999	7.8	79.6	9.8		104.7	1.2		102.9	0.3
11	atrazine-desethyl	0.1	-100	0.03	0.1	0.9999	ND	66.0	10.4		94.2	1.6		92.6	0.6
12	atrazine-desisopropyl	5	-100	1.5	5.0	0.9976	11.5	76.6^*^	18.6		82.2	10.6		74.7	9.5
13	beauvericin	0.1	-100	0.03	0.1	0.9968	ND	96.3	4.1		103.3	7.6		81.1	7.0
14	benfuracarb	0.1	-100	0.03	0.1	0.9988	ND	89.2	1.9		98.2	6.4		92.2	3.0
15	benzovindiflupyr	0.1	-100	0.03	0.1	0.9954	ND	69.9	14.8		109.5	11.2		89.9	7.2
16	buprofezin	0.1	-100	0.03	0.1	0.9999	ND	103.3	3.4		115.6	3.6		111.6	2.0
17	butachlor	0.5	-100	0.15	0.5	0.9999	ND	81.6	1.9		96.5	2.2		90.3	1.1
18	cadusafos	0.1	-100	0.03	0.1	0.9999	ND	78.5	2.2		115.5	2.8		100.7	3.4
19	carbaryl	0.5	-100	0.15	0.5	0.9976	ND	68.4	7.0		96.6	2.2		90.1	0.8
20	carbendazim	0.1	-100	0.03	0.1	0.9997	0.3	88.0	2.1		93.7	0.8		91.7	0.3
21	carbofuran	0.1	-100	0.03	0.1	0.9995	ND	83.7	1.8		94.9	1.0		92.5	0.9
22	chlorobenzuron	0.5	-100	0.15	0.5	0.9998	ND	89.1	3.7		95.1	1.4		93.7	1.0
23	chlorotoluron	0.1	-100	0.03	0.1	0.9997	ND	84.9	2.1		97.0	1.1		95.8	1.3
24	chlorpyrifos	0.5	-100	0.15	0.5	0.9993	ND	84.9	3.2		97.6	2.0		94.5	1.2
25	chlorpyrifos-methyl	1	-100	0.3	1.0	0.9994	ND	80.1	9.9		90.2	2.0		87.7	1.0
26	chlorsulfuron	0.2	-100	0.06	0.2	0.9995	ND	61.1	8.5		67.8	5.1		62.4	3.6
27	clothianidin	1	-100	0.3	1.0	0.9989	ND	85.6	5.6		87.7	0.7		82.6	1.5
28	coumaphos	0.5	-100	0.15	0.5	0.9996	ND	97.0	2.7		105.3	2.3		100.5	1.2
29	cyanazine	0.1	-100	0.03	0.1	0.9999	ND	95.5	2.4		98.3	1.0		96.3	0.3
30	demeton O & S	1	-100	0.3	1.0	0.9996	ND	92.5	5.8		95.4	2.0		95.1	1.9
31	demeton-S-methyl sulfone	1	-100	0.3	1.0	0.9991	ND	79.9	5.7		97.1	1.8		92.6	1.0
32	demeton-S-sulfoxide	0.5	-100	0.15	0.5	0.9998	ND	99.4	2.2		108.3	1.5		103.7	0.8
33	dichlorvos	0.5	-50	0.15	0.5	0.9993	ND	71.2	2.1		66.1	1.3		68.3	3.0
34	diflubenzuron	0.5	-100	0.15	0.5	0.9998	ND	81.9	8.4		85.8	2.7		87.8	2.1
35	dimethoate	0.5	-100	0.15	0.5	0.9998	ND	75.4	2.9		84.4	1.0		82.5	1.3
36	dinocap	5	-100	1.5	5.0	0.9976	ND	88.2^*^	5.5		88.5	3.3		97.3	8.2
37	dinoseb	5	-100	1.5	5.0	0.9967	ND	63.6^*^	4.3		90.0	8.3		89.3	2.5
38	disulfoton-sulfone	0.5	-100	0.15	0.5	0.9990	ND	66.3	3.7		88.7	1.6		84.4	0.7
39	disulfoton-sulfoxide	0.2	-100	0.06	0.2	0.9992	ND	94.0	4.1		110.7	2.5		107.1	0.8
40	diuron	0.5	-100	0.15	0.5	0.9996	ND	83.8	3.2		93.8	1.6		91.0	1.4
No.	Pesticide	Linear range/ (ng/L)		LOD/ (ng/L)	LOQ/ (ng/L)	r^2^	Raw water								
							Content/ (ng/L)	Spiked at 1 ng/L			Spiked at 20 ng/L			Spiked at 50 ng/L	
								Recovery/ %	RSD/ %		Recovery/ %	RSD/ %		Recovery/ %	RSD/ %
41	EPN	0.5	-100	0.15	0.5	0.9994	ND	61.0	6.9		66.3	2.8		61.1	4.5
42	fenamiphos	0.1	-100	0.03	0.1	0.9974	ND	81.0	6.6		118.1	2.1		109.8	2.3
43	fenamiphos sulfone	0.5	-100	0.15	0.5	0.9997	ND	90.5	3.8		93.4	1.3		92.2	1.8
44	fenamiphos sulfoxide	0.5	-100	0.15	0.5	0.9998	ND	92.9	2.2		98.1	1.2		95.3	0.8
45	fenobucarb	0.1	-100	0.03	0.1	0.9998	ND	93.2	2.8		99.8	0.9		97.4	1.6
46	fenthion	1	-100	0.3	1.0	0.9995	ND	95.6	7.6		95.4	1.5		92.8	2.0
47	fenthion sulfoxide	0.5	-100	0.15	0.5	0.9998	ND	95.5	3.1		98.5	1.5		95.1	1.1
48	fenthion-sulfone	1	-100	0.3	1.0	0.9996	ND	75.8^*^	8.8		79.0	2.8		76.6	1.4
49	fipronil	1	-100	0.3	1.0	0.9995	ND	83.3	14.4		96.3	3.3		85.8	2.9
50	fipronil desulfinyl	0.5	-100	0.15	0.5	0.9999	ND	94.6	8.0		95.5	2.2		90.2	2.3
51	fipronil sulfide	1	-100	0.3	1.0	0.9993	ND	71.7	10.6		95.5	2.8		85.5	1.9
52	fipronil-sulfone	0.5	-100	0.15	0.5	0.9992	ND	84.8	5.1		96.5	4.2		86.7	2.5
53	fonofos	0.1	-100	0.03	0.1	0.9998	ND	91.1	2.4		97.4	1.2		96.3	0.7
54	fosthiazate	0.1	-100	0.03	0.1	0.9991	ND	84.8	1.7		105.4	2.2		101.3	1.3
55	3-hydroxycarbofuran	0.5	-100	0.15	0.5	0.9960	ND	94.2	2.4		100.4	3.5		93.2	1.9
56	imidacloprid	0.5	-100	0.15	0.5	0.9998	4.1	74.0	2.8		76.5	1.4		74.2	1.3
57	imidacloprid-urea	0.1	-100	0.03	0.1	0.9999	ND	73.1	13.3		63.3	13.2		70.3	16.2
58	imidaclothiz	1	-100	0.3	1.0	0.9998	ND	73.4	3.9		77.5	2.3		74.6	0.7
59	iprobenfos	0.1	-50	0.03	0.1	0.9996	ND	78.5	1.3		111.8	2.3		100.3	2.1
60	isoprocarb	0.1	-100	0.03	0.1	0.9997	ND	78.0	4.4		84.7	1.1		81.6	2.8
61	isoprothiolane	0.1	-100	0.03	0.1	0.9993	ND	77.6	2.4		103.3	2.1		95.3	1.0
62	isoproturon	0.1	-100	0.03	0.1	0.9999	ND	88.8	1.8		100.0	1.3		97.8	0.6
63	malaoxon	0.1	-100	0.03	0.1	0.9988	ND	94.3	2.7		119.8	1.0		114.6	0.7
64	malathion	0.1	-100	0.03	0.1	0.9990	ND	73.1	4.9		102.4	2.1		93.7	0.9
65	mefenacet	0.1	-100	0.03	0.1	0.9999	ND	79.5	1.7		96.8	1.3		93.3	0.8
66	mepronil	0.1	-100	0.03	0.1	0.9999	ND	65.1	1.6		78.8	1.1		78.3	0.7
67	metalaxyl	0.1	-100	0.03	0.1	0.9992	ND	96.7	4.2		102.7	2.2		99.7	0.9
68	methidathion	0.5	-100	0.15	0.5	0.9995	ND	75.2	2.4		86.2	2.1		82.2	2.6
69	metolachlor	0.1	-100	0.03	0.1	0.9998	ND	84.7	1.4		98.5	1.0		96.5	0.9
70	metsulfuron-methyl	0.1	-100	0.03	0.1	0.9993	ND	80.2	2.4		97.6	1.3		89.2	1.5
71	molinate	0.1	-100	0.03	0.1	0.9998	ND	91.2	2.7		99.2	1.5		98.4	0.8
72	monocrotophos	0.2	-100	0.06	0.2	0.9999	ND	86.9	4.0		96.0	1.0		93.3	1.0
73	nitenpyram	5	-100	1.5	5.0	0.9967	ND	103.2^*^	6.3		82.6	4.0		76.0	6.0
74	oxamyl	1	-100	0.3	1.0	0.9978	ND	67.7	13.2		74.2	13.7		72.3	8.7
75	parathion	1	-100	0.3	1.0	0.9995	ND	62.1	7.3		88.8	3.8		89.9	3.1
76	parathion-methyl	5	-100	1.5	5.0	0.9987	ND	61.6^*^	2.5		75.0	6.3		73.3	6.1
77	pencycuron	0.1	-100	0.03	0.1	0.9998	ND	81.1	2.3		95.4	1.2		91.4	1.1
78	pendimethalin	0.2	-100	0.06	0.2	0.9991	ND	87.0	2.1		94.8	2.1		92.8	1.1
79	phenthoate	0.5	-100	0.15	0.5	0.9977	ND	90.9	7.4		95.3	2.5		90.9	1.0
80	phorate	0.1	-100	0.03	0.1	0.9996	ND	85.5	1.5		94.6	2.0		90.4	1.0
81	phorate sulfone	1	-100	0.3	1.0	0.9996	ND	82.9	3.8		84.9	1.3		82.6	2.6
82	phorate sulfoxide	0.5	-100	0.15	0.5	0.9994	ND	86.3	3.4		104.9	1.1		101.1	0.4
83	phosfolan-methyl	0.5	-100	0.15	0.5	0.9996	ND	81.4	4.5		86.6	2.8		83.9	1.2
84	phosphamidon	0.1	-100	0.03	0.1	0.9995	ND	89.7	0.9		100	1.3		97.8	0.9
85	phoxim	0.5	-100	0.15	0.5	0.9993	ND	82.3	3.0		89.6	2.3		82.5	2.2
86	pretilachlor	0.1	-100	0.03	0.1	0.9997	ND	63.2	3.1		82.7	2.4		70.9	1.6
87	procymidone	1	-100	0.3	1.0	0.9956	ND	99.9	7.1		103.3	3.1		98.8	2.4
88	pymetrozin	0.1	-100	0.03	0.1	0.9999	ND	114.2	2.7		107.2	3.7		106.6	3.7
89	pyridaben	0.1	-50	0.03	0.1	0.9990	ND	89.6	1.4		71.2	3.6		78.9	5.5
No.	Pesticide	Linear range/ (ng/L)		LOD/ (ng/L)	LOQ/ (ng/L)	r^2^	Raw water								
							Content/ (ng/L)	Spiked at 1 ng/L			Spiked at 20 ng/L			Spiked at 50 ng/L			
								Recovery/ %	RSD/ %		Recovery/ %	RSD/ %		Recovery/ %	RSD/ %
90	rotenone	0.5	-100	0.15	0.5	0.9982	ND	83.5	5.3		109.3	1.0		95.9	1.2
91	simazine	0.2	-100	0.06	0.2	0.9999	ND	98.6	1.9		100.1	1.7		99.1	0.9
92	simetryn	0.1	-100	0.03	0.1	0.9999	ND	99.3	1.1		102.7	0.9		100.2	0.6
93	sulfotep	0.5	-100	0.15	0.5	0.9994	ND	73.3	3.9		90.6	2.7		82.2	2.4
94	tebuconazole	0.1	-100	0.03	0.1	0.9998	0.7	84.3	2.8		95.3	1.7		93.5	0.6
95	terbufos	1	-100	0.3	1.0	0.9980	ND	60.6	6.4		95.8	4.3		86.7	2.6
96	terbufos sulfone	1	-100	0.3	1.0	0.9995	ND	69.4	11.3		101.3	1.9		98.8	1.6
97	terbufos sulfoxide	0.1	-100	0.03	0.1	0.9993	ND	81.1	2.6		99.5	1.5		96.9	0.7
98	terbuthylazine	0.1	-100	0.03	0.1	0.9999	ND	101.5	0.7		104.4	0.9		102.6	0.6
99	thiabendazole	1	-100	0.3	1.0	0.9996	ND	73.3	5.0		75.4	2.2		90.8	2.7
100	thiacloprid	0.2	-100	0.06	0.2	0.9998	ND	81.9	2.6		86.1	0.7		84.3	1.5
101	thiamethoxam	1	-100	0.3	1.0	0.9992	ND	76.7	5.6		80.5	2.0		80.3	3.3
102	thiodicarb	0.1	-100	0.03	0.1	0.9991	ND	93.6	2.6		118.9	2.9		115.0	1.2
103	triadimefon	0.1	-100	0.03	0.1	0.9999	ND	88.5	2.2		94.7	2.8		91.2	1.8
104	triazophos	0.2	-100	0.06	0.2	0.9998	ND	85.7	2.1		99.1	2.6		96.3	1.0
105	tricyclazole	0.1	-100	0.03	0.1	0.9999	ND	84.2	1.8		90.8	1.5		88.8	1.3
106	triflumuron	0.2	-100	0.06	0.2	0.9999	ND	93.5	3.3		101.9	3.3		98.7	0.5
107	trifluralin	1	-100	0.3	1.0	0.9996	ND	96.6	5.9		92.5	2.9		91.6	1.7

ND: not detected. * Spiked at 5 ng/L.

#### 2.6.2 回收率和精密度

仪器进样分析之前,应做空白试验,对实验用水质量、试剂纯度、器皿洁净度等方面进行考察,本底污染应该控制在低于方法检出限;在测定高浓度样品后为防止交叉污染或残留干扰,应进行空白样品测试。

分别向饮用水及水源水中添加低、中、高3个水平(1、20、50 ng/L)的标准溶液进行加标回收试验,每个水平平行进行6组,计算回收率及相对标准偏差(RSD),结果见表4。饮用水中107种目标分析物在3个水平下的加标回收率(*n*=6)分别为61.4%~91.4%、62.8%~119.0%和61.2%~115.7%,RSD分别为0.9%~17.1%、0.6%~14.1%和0.4%~12.7%。水源水中107种目标分析物在3个水平下的加标回收率(*n*=6)分别为60.6%~94.2%、61.1%~119.8%和61.1%~115%,RSD分别为0.7%~18.6%、0.7%~15.7%和0.3%~16.2%。

### 2.7 实际样品的测定

用本方法分别对水源水及饮用水开展107种农药及其代谢产物检测,其中水源水20件,饮用水20件。107种目标分析物在水源水中检出42种,浓度水平在0.1~97.1 ng/L,饮用水中检出32种,浓度水平在0.1~93.6 ng/L,酰胺类除草剂、三嗪类除草剂、三唑类杀菌剂、烟碱类杀虫剂、氨基甲酸酯类杀虫剂等检出率较高,检出种类、浓度见表5。

**表5:** 水源水和饮用水中农药的主要检出情况

Class	Pesticides	Raw water (n=20)					Drinking water (n=20)			
		Detection rate/%		Content range/(ng/L)			Detection rate/%		Content range/(ng/L)	
Carbamate insecticides	benfuracarb, carbofuran, aldicarb sulfone, isoprocarb	5	-90	0.1	-5.5		10	-55	0.1	-10.2
Sulfonylurea herbicides	chlorsulfuron, metsulfuron-methyl	25	-50	0.1	-1.4		0	-35	ND	-0.5
Triazine herbicides	atrazine, atrazine-2-hydroxy, atrazine-desethyl, atrazine-desisopropyl, cyanazine, simazine, simetryn, terbuthylazine	50	-100	0.1	-97.1		0	-100	ND	-93.6
Triazole insecticides/ fungicides	triazophos, tebuconazole, triadimefon, tricyclazole,	40	-100	0.1	-65.2		0	-95	ND	-64.6
Amide herbicides	acetochlor, butachlor, metolachlor, pretilachlor	75	-100	0.1	-37.6		50	-85	0.1	-26.8
Neonicotinoid insecticides	acetamiprid, clothianidin, imidacloprid, imidacloprid- urea, thiacloprid, thiamethoxam	35	-100	0.1	-95.8		15	-85	0.1	-78.4

ND: not detected.

### 2.8 与其他方法对比

将本方法与水体中农药类相关标准检验方法进行了对比,本方法样品用量少、前处理简单快速,样品富集及分析时长仅23 min,可同时对107种典型农药及代谢产物进行测定,检测效率优于其他方法,见表6。离线SPE法或液液萃取法,需将目标分析物提取、氮吹、复溶,仅少量复溶液进质谱检测,在线SPE法中样品富集后全部洗脱至质谱检测,因此富集倍数高,所需有机试剂种类少、用量小,准确灵敏,更适合于水体中痕量农药测定。相较于离线SPE柱的一次性使用,在线SPE柱可反复使用,本方法中SPE柱累计使用次数约为450次,通过对不同批次测定的农药类化合物保留时间、峰形及峰面积进行比较,无显著性差异。

**表6:** 本方法与其他方法的比较

Detection method	Samples	Number of detected pesticides	LODs	Sample volume/ mL	Reagents	Ref.
Online-SPE-LC-MS/MS	raw water, drinking water	107	0.03-1.5 ng/L	5	acetonitrile	this study
SPE-LC-MS/MS	drinking water	450 (including 7 groups)	0.01-65 mg/L	25	acetonitrile, methylbenzene, sodium acetate, anhydrous sodium sulfate, anhydrous magnesium sulfate	[16]
SPE-LC-MS/MS	surface water, groundwater, domestic sewage, industrial waste water	15	0.002-0.031 μg/L	100	methanol	[17]
Liquid-liquid extraction- LC-MS/MS	surface water, groundwater	40	0.1-5 μg/L (LOQ)	20	acetone, dichloromethane, hexane, acetonitrile, anhy- drous sodium sulfate	[18]

## 3 结论

本研究建立了online-SPE-UPLC-MS/MS快速筛查和确证饮用水中107种典型农药及代谢产物的方法,可对水体中有机磷类、氨基甲酸酯类、酰胺类、苯甲酰脲类、新烟碱类等农药及代谢产物同时测定,具有高通量、高灵敏度、高准确度的优点,实际应用价值较高。使用该方法对水源水及饮用水进行检测发现,水体中农药类物质复合污染情况较为普遍,除了需要对水体中农药类指标检测外,对其代谢产物污染水平研究有待进一步开展,以提供全面、准确的检测数据,为水环境农药污染监测及饮水安全提供技术支撑。
